# From Awareness to Action: Women’s Self-Care Strategies and Clinical Behaviors in Recurrent Urinary Tract Infections

**DOI:** 10.3390/medicina62020295

**Published:** 2026-02-02

**Authors:** Laura Miszewska, Kevin Miszewski, Bartłomiej Marczak, Gabriela Kucko, Marcin Matuszewski

**Affiliations:** 1 Faculty of Medicine, Medical University of Gdańsk, ul. Smoluchowskiego 17 UCK, 80-214 Gdańsk, Poland; 2 Department of Urology, Medical University of Gdańsk, ul. Smoluchowskiego 17 UCK, 80-214 Gdańsk, Poland

**Keywords:** recurrent urinary tract infection, cystitis, women’s health, antibiotic stewardship, non-antibiotic prophylaxis, patient-reported outcomes

## Abstract

*Background and Objectives:* Recurrent urinary tract infection (rUTI) remains common and burdensome, with growing emphasis on antibiotic stewardship and non-antibiotic prevention. We characterized what women with rUTI know, do, and receive in everyday care and identified gaps between patient understanding and guideline-concordant management. *Materials and Methods:* We conducted a single-center, cross-sectional survey of consecutive adult women presenting with rUTI to a urology clinic in Poland. A structured questionnaire captured demographics, knowledge, symptoms and triggers, diagnostics, treatments and prevention, and satisfaction. Responses were standardized a priori; descriptive statistics and exploratory comparisons were performed (N = 36). *Results:* The mean age was 53.0 years (SD: 14.8). Only 36.1% identified the correct rUTI definition, while 83.3% recognized bacteria as the common cause. The symptom profile was dominated by frequency and dysuria (each 88.9%); 27.8% reported intercourse as a precipitant, and this was more frequent among sexually active women (43.5% vs. 7.7%; *p* = 0.031). Over half (55.6%) perceived no seasonality. The median number of episodes in the prior year was five (IQR 4–6). Urine culture was obtained before treatment in 38.9% and after treatment in 13.9%. The overall satisfaction with care was low to moderate (13.9% were very satisfied, 61.1% were moderately satisfied, and 25.0% were dissatisfied). Prior antibiotic exposure included ciprofloxacin (55.6%), furazidin (75.0%), and fosfomycin (47.2%). The uptake of preventive options was uneven: immunoactive vaccines accounted for 19.4%, methenamine hippurate for 16.7%, pelvic floor physiotherapy for 33.3%, and vaginal estrogen for 5.6% overall (9.5% among women ≥ 50 years). *Conclusions:* In this clinic-referred cohort, rUTI was frequent and disruptive, factual knowledge was limited, urine culture use was inconsistent, and fluoroquinolone exposure remained common. Preventive care was misaligned with guidelines, with underuse of vaginal estrogen and variable adoption of non-antibiotic strategies. Targeted education, stewardship, and structured access to evidence-based prevention may improve outcomes.

## 1. Introduction

Urinary tract infection (UTI) is the most common bacterial infection in women: approximately 50 to 60% experience at least one episode during their lifetime, and 20–30% of those affected develop a recurrence within a year, meeting the definition of recurrent UTI (rUTI: ≥2 episodes in 6 months or ≥3 in 12 months) [1]. Although most acute cystitis episodes are self-limited, the cumulative burden of rUTI is substantial, accounting for millions of outpatient visits and more than US $1 billion in direct costs annually [2]. Beyond healthcare utilization, rUTI impairs multiple dimensions of well-being. Studies report increased anxiety, depressive symptoms, sexual distress, and social withdrawal among affected women, many of whom describe “living life around the next infection” [3,4]. In both primary care and urological settings, clinicians also report frustration with the recurring cycle of urine testing, short-course antibiotics, and rapid relapse, which may contribute to patient dissatisfaction and repeat consultations [5]. International guidelines now emphasize antibiotic stewardship and shared decision making. The 2024 update of the European Association of Urology (EAU) Guidelines recommends targeted therapy, discourages treatment of asymptomatic bacteriuria, and highlights non-antibiotic prevention strategies such as increased hydration and vaginal estrogen in postmenopausal women [2]. The 2022 AUA guideline similarly advocates the shortest effective antibiotic course and individualized prophylaxis strategies [6]. However, rising resistance among common uropathogens, particularly Escherichia coli, is undermining the effectiveness of several agents traditionally used in practice, including fluoroquinolones and trimethoprim–sulfamethoxazole [7]. In parallel with stewardship efforts, interest in non-antibiotic approaches has increased. Moderate-strength evidence supports cranberry products for risk reduction [8]. For D-mannose, earlier randomized trials and pooled analyses suggested a potential benefit in reducing recurrence rates, particularly in women with uncomplicated rUTI. In a 2014 randomized controlled trial, D-mannose demonstrated comparable effectiveness to nitrofurantoin prophylaxis with fewer adverse effects, while a subsequent meta-analysis reported a reduction in recurrence risk compared with placebo or no prophylaxis [9].

However, more recent high-quality evidence has challenged these findings. A large randomized clinical trial published in 2024 found that six months of daily D-mannose did not reduce recurrent, medically attended UTIs compared with placebo and, therefore, should not be recommended for routine prophylaxis [10]. Taken together, these data suggest that earlier signals of efficacy may not translate into clinically meaningful benefit in contemporary, pragmatic settings, and they highlight the need to contextualize D-mannose use within shared decision making and evolving evidence. Additional options under investigation include immunoactive preparations such as MV140 [11] and multimodal behavioral programs incorporating pelvic floor physiotherapy, timed voiding, and fluid optimization [12]. Nevertheless, uptake in routine care remains uneven; analyses of patient advocacy and social media discussions highlight uncertainty regarding evidence strength, inconsistent clinician advice, and a perception that management often proceeds by trial and error [13]. Despite the expanding evidence base, limited data describe what women with rUTI understand, believe, and do when managing their condition, particularly among those seeking specialist care. Many existing surveys rely on community samples or online panels rather than patients presenting to urology clinics after extended care pathways. To address this gap, we developed a comprehensive questionnaire covering demographics, knowledge, symptom triggers, antibiotic experiences, and awareness of non-prescription strategies, and administered it to women attending our urology practice after often prolonged journeys through primary and secondary care. We aimed to describe what women with rUTI know, do, and receive in everyday care. Specifically, we assessed the understanding of causes, risk factors, and evidence-based prevention; documented self-care practices and clinical behaviors, including the use of recommended investigations and therapies; and explored discrepancies between patient beliefs and contemporary best practice. Given that our sample was recruited in a specialist urology setting, our findings are intended to characterize patient experience within a referral population, rather than to estimate population-level prevalence of behaviors. By linking knowledge with actions and care received, this study aims to inform patient education and service improvements that support person-centered, evidence-aligned management of this common and persistent condition.

## 2. Materials and Methods

### 2.1. Study Design and Setting

We conducted a single-center, cross-sectional survey in a urology practice. Consecutive adult women presenting with rUTI were invited to participate. Most participants had longstanding symptoms and had consulted primary or secondary care services before referral to our clinic. As a consequence, the cohort likely represents a more refractory, specialist-referred subgroup, which may differ systematically from women managed solely in primary care.

### 2.2. Participants and Eligibility Criteria

The eligibility criteria were female sex, age ≥ 18 years, a history of cystitis, and the ability to complete a Polish-language questionnaire. Participation was voluntary and anonymous, and all returned questionnaires were analyzed (N = 36).

### 2.3. Ethical Approval and Informed Consent

The study protocol and informed-consent documents were approved by the Institutional Review Board of the Medical University of Gdańsk. Written informed consent was obtained from all participants prior to enrolment. This study was conducted in accordance with the Declaration of Helsinki.

### 2.4. Data Collection

All data were collected by patient self-report at the time of the visit. Because many participants were newly referred and had received prior care across multiple external settings, medical records were not consistently available; therefore, no systematic verification against clinical documentation was performed. This design increases the risk of recall inaccuracy, particularly for prior antibiotic exposures and diagnostic practices, and may lead to underestimation or overestimation of reported proportions. Data were collected using a Polish questionnaire. The instrument captured demographics; sexual and obstetric history; knowledge items covering the definition of rUTI, etiology, risk factors, and non-pharmacological strategies; current symptoms, perceived triggers, and seasonality; diagnostic practices, including pre- and post-treatment urine cultures and prior cystoscopy; antibiotic exposure and prophylaxis; and satisfaction with care. For transparency and reproducibility, an English-language version of the questionnaire was prepared by the study team. The translated questionnaire is provided as Supplementary Material.

### 2.5. Data Processing

Free-text entries for antibacterial agents were harmonized to international non-proprietary names (for example, furazidin, fosfomycin, and ciprofloxacin) to enable consistent analysis. Key variables were prespecified. Knowledge accuracy was judged against guideline-concordant responses for the rUTI threshold and for bacterial etiology. Age ≥50 years was used as a pragmatic proxy for postmenopausal status. Responses were standardized before analysis: numerical ranges or free text were converted to single numeric values where applicable, multi-select items were recoded as binary indicators, and urine culture practices were classified as pre-treatment or post-treatment. Patient satisfaction was recorded on a three-level ordinal scale (very satisfied, moderately satisfied, or dissatisfied).

### 2.6. Statistical Analysis

We described participant characteristics and survey responses entered into an electronic database using means (standard deviation (SD)) or medians (interquartile range (IQR)) based on their distribution. Because between-group comparisons were exploratory and this study was not powered for hypothesis testing, we report *p*-values as descriptive and interpret them cautiously. To address multiplicity, we additionally applied a Holm–Bonferroni adjustment for the main set of exploratory comparisons reported in the Results. A *p*-value of <0.05 was considered statistically significant for unadjusted analyses; adjusted results are presented to contextualize false-positive risk. Statistical analysis was performed using IBM SPSS Statistics for Windows, version 29 (IBM Corp., Armonk, NY, USA).

## 3. Results

### 3.1. Patient Demographics and Cohort Characteristics

Following screening, 36 women were included in the analysis. The mean age was 53.0 years (SD: 14.8). Detailed demographic and baseline clinical characteristics are presented in Table 1. In total, 47.2% of participants reported tertiary education, and 22.2% lived in urban areas of ≥100,000 inhabitants. A majority were sexually active (63.9%) and parous (91.7%), with 25.0% using any form of contraception. Regular sport was reported by 25.0%.

### 3.2. Knowledge and Awareness

Overall, knowledge showed mixed accuracy. In total, 36.1% identified the correct definition of recurrent UTI (≥3/year), while 83.3% recognized bacteria as the most common cause. Awareness of non-pharmacologic measures was uneven: cranberry products (79.4%), post-coital voiding (55.9%), and urogynecology physiotherapy (55.9%) were most frequently endorsed. In exploratory analyses, formal educational attainment did not translate into more accurate knowledge of the rUTI definition: women with tertiary education were no more likely than others to identify the correct threshold (*p* = 0.721).

### 3.3. Symptoms, Triggers, and Seasonality

**Figure 1:**
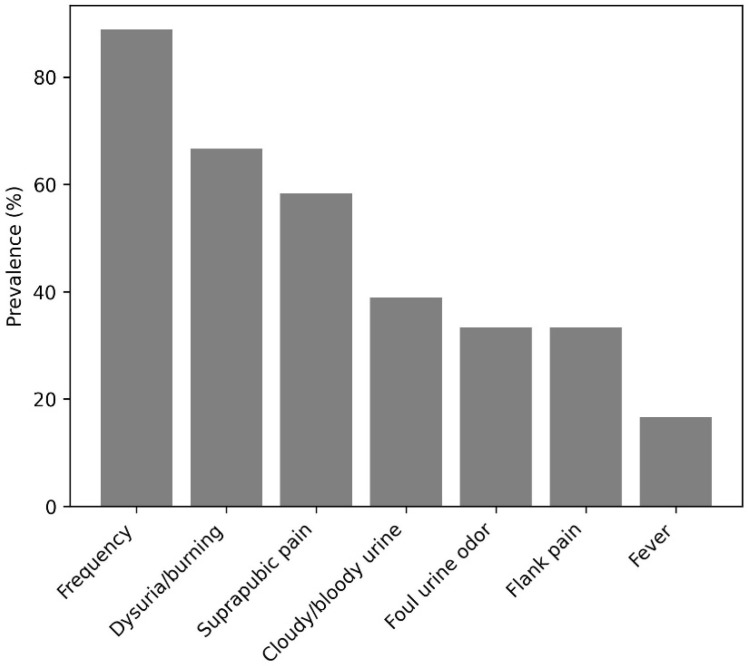
Prevalence of lower urinary tract symptoms in women with rUTI.

**Figure 2:**
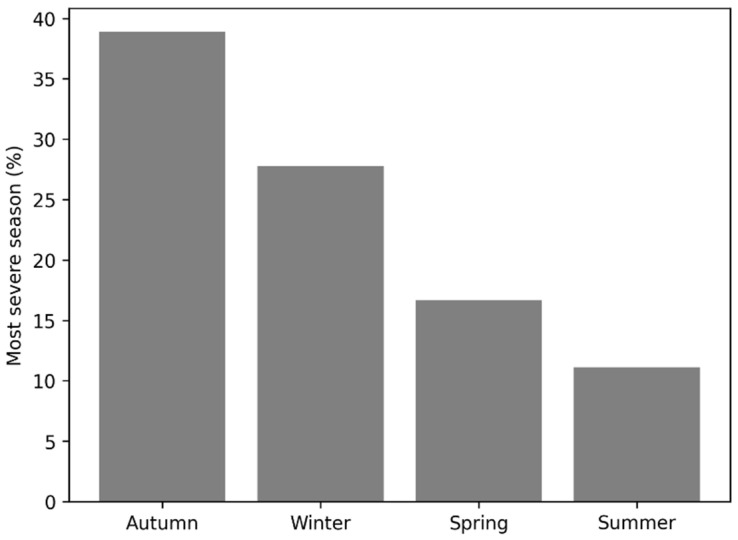
Most affected season reported by women with rUTI.

Lower urinary tract symptoms were dominated by frequency (88.9%) and dysuria (88.9%), with suprapubic pain also common (58.3%) (Figure 1). Cloudy or bloody urine was reported by 38.9%, foul urine odor by 33.3%, and flank pain by 27.8%; fever was less frequent (13.9%). Regarding precipitants, cold exposure/chill was the most frequently cited trigger (66.7%), followed by post-coital/sexual activity (27.8%). Consistent with this pattern, sexually active participants were more likely to report intercourse as a precipitating factor than those not sexually active (43.5% vs. 7.7%; *p* = 0.031). With respect to seasonality, 55.6% reported no consistent seasonal pattern. Among those who perceived seasonality (n = 16), symptoms most often worsened in autumn (75.0%) and winter (50.0%), with spring (25.0%) and summer (18.8%) cited less often (Figure 2).

### 3.4. Symptom Burden and Care Pathways

Participants reported a high burden of recurrence, with a median of five episodes in the preceding year. In terms of diagnostic practice, urine culture before treatment was obtained by 38.9%, and post-treatment culture by 13.9%. For the most recent episode, respondents most often reported a positive urine culture (52.8%), with accompanying leukocyturia (33.3%), hematuria (19.4%), and infrequent proteinuria (2.8%). Cystoscopy had been performed in 13.9% overall. Satisfaction with current management was 61.1% for “moderately satisfied,” 13.9% for “very satisfied,” and 25.0% for dissatisfied. Notably, routine pre-treatment urine culture did not correspond to higher patient satisfaction (*p* = 0.627).

### 3.5. Impact on Daily Functioning

**Figure 3:**
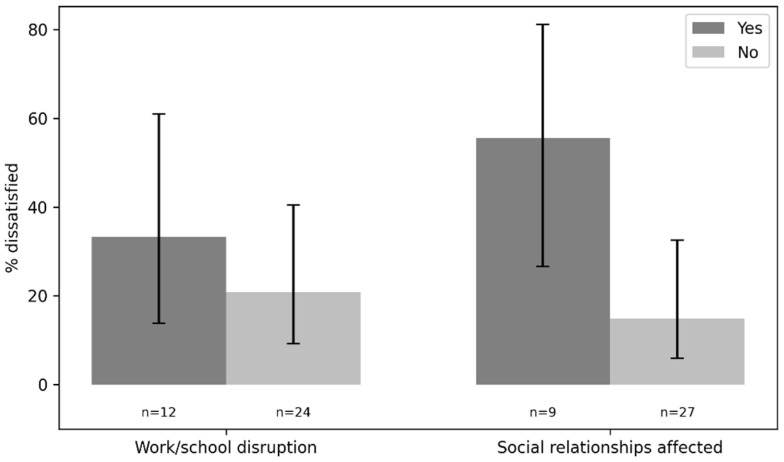
Proportion of patients dissatisfied with care by reported life impact.

Cystitis episodes substantially disrupted daily life. The most frequently affected domains were physical activity and sleep, and the most recent episode lasted a median of 6 days (IQR: 4–7). Impact on social relationships was strongly associated with dissatisfaction with care: among women reporting social impact, 55.6% were dissatisfied versus 14.8% among those without social impact (*p* = 0.026) (Figure 3). Dissatisfied respondents also reported more recurrences in the prior year (median: 6.0 vs. 4.0; *p* = 0.043). Work/school disruption was more common in younger women (median age: 46.0 vs. 56.5 years; *p* = 0.014), suggesting a different burden profile by life stage.

### 3.6. Antibiotic Exposure and Tolerability

Historically used agents were heterogeneous, with fluoroquinolone exposure notably common. Ciprofloxacin was reported by 55.6%, alongside furazidin (nitrofuran) (75.0%) and fosfomycin (47.2%). Additional agents included amoxicillin/clavulanate (25.0%) and trimethoprim–sulfamethoxazole (22.2%). Levofloxacin was infrequently reported (2/36; 5.6%), norfloxacin was not reported, and use of pivmecillinam was rare (1/36; 2.8%). Of non-antibiotic urinary antiseptics, methenamine hippurate was reported by 16.7%, typically in a preventive context. The average treatment duration was 9.4 days, and 69.4% stated they routinely completed the full prescribed course. Any self-reported antibiotic-related adverse effect occurred in 5.6% of respondents. Notably, nitrofurantoin was seldom used; in our setting, the nitrofuran agent most commonly prescribed is furazidin, which is regionally marketed and occupies a role analogous to nitrofurantoin in Anglo-American practice.

### 3.7. Prevention Strategies and Other Therapies

Use of preventive strategies favored antibiotics: prophylactic furazidin (13/36 (36.1%)) and fosfomycin (9/36 (25.0%)) were common. Immunoactive vaccines (7/36 (19.4%)) were reported. Pelvic floor physiotherapy accounted for 12/36 (33.3%), while intravesical hyaluronic acid (1/36 (2.8%)) was rare. Vaginal estrogen (vaginal globules/creams) was used by 2/36 (5.6%) overall and 2/21 (9.5%) among women aged ≥50; additionally, vaginal probiotics/prebiotics were reported by 5/36 (13.9%). Among bladder-directed or adjunctive therapies, solifenacin had been used by 9/36 (25.0%), while mirabegron and oxybutynin were each reported by 1/36 (2.8%).

## 4. Discussion

In this specialty-referred cohort of women with rUTI, we observed a high symptomatic burden, substantial interference with daily functioning, and areas of divergence between evidence-based recommendations and reported care. To contextualize these findings against contemporary recommendations, we summarized key areas of alignment and divergence between reported practices and EAU/AUA guideline-concordant care in Table 2. Although this study was conducted in a single center and included a limited number of participants, the cohort reflects a clinically relevant subgroup of women with rUTI who experience persistent symptoms and repeated healthcare encounters prior to specialist referral. A median of five episodes per year, together with sleep disruption and reduced activity, markedly diminishes quality of life (QoL). These findings align with contemporary patient reports and underscore the value of condition-specific QoL tools in routine care [3,14,15]. A notable finding was limited factual knowledge: only one-third of respondents correctly identified the guideline definition of rUTI. Such confusion is well documented and can impede shared decision making and timely access to appropriate prevention [16]. In our analysis, sexually active participants were more likely to report intercourse as a precipitating factor than those not sexually active (43.5% vs. 7.7%; *p* = 0.031), indicating a strong association. This is consistent with evidence that intercourse-related rUTI is common and can be mitigated with tailored measures such as postcoital antibiotic prophylaxis and patient-initiated self-start therapy [17,18]. Several areas where reported practice diverged from guideline recommendations were observed. First, vaginal estrogen, a strongly endorsed and low-risk intervention for hypoestrogenic women, was underused, with only about 10% of women aged 50 years or older receiving it. A large cohort study showed a >50% reduction in UTIs after prescription of vaginal estrogen [19], while randomized trials demonstrated clinically meaningful benefit versus placebo [20]. Both AUA and EAU guidelines recommend vaginal estrogen for rUTI prevention in postmenopausal women [2,6]. Second, antimicrobial stewardship warrants attention. More than half of the cohort reported prior ciprofloxacin exposure despite guidance to reserve fluoroquinolones for selected scenarios, given rising resistance and important safety concerns (e.g., tendinopathy, neuropathy, and aortic risk) [21]. Safer first-line agents, including nitrofurantoin (or nitrofuran alternatives) and fosfomycin, where available, should be prioritized in line with current recommendations. Third, our respondents reported low rates of urine culture before treatment and very low rates after treatment. Guidelines advise obtaining a culture in recurrent episodes or when resistance is suspected, while avoiding screening or treating asymptomatic bacteriuria except in defined circumstances (e.g., pregnancy, and prior to mucosal urologic procedures) [2]. This approach improves diagnostic precision and supports antimicrobial stewardship without promoting unnecessary testing. Use of non-antibiotic prevention in our cohort was inconsistent. Methenamine hippurate was used by 16.7%, aligning with the ALTAR randomized trial showing non-inferiority to daily antibiotic prophylaxis over 12 months [22]. Immunoactive vaccines were used by 19.4%; early studies of the sublingual polybacterial vaccine MV140 suggest benefits, although long-term effectiveness and regulatory status remain uncertain [10,23]. In contrast, cranberry products were widely recognized (with awareness at 79.4%) yet rarely used; clearer counselling on product selection and dosing is warranted, in line with the 2023 Cochrane review [8]. Overall, these patterns suggest that specialist care is already incorporating some non-antibiotic options (methenamine and immunoprophylaxis), but there remains room to translate awareness into effective use for cranberry and to de-implement strategies lacking benefit, while reinforcing guideline-concordant choices. These observations are further supported by recent microbiome-oriented evidence suggesting that rUTI is linked to persistent dysbiosis of the urinary and vaginal microbiota. In this context, frequent or prolonged antibiotic use may perpetuate microbial imbalance and susceptibility to recurrence, whereas preventive strategies that limit antibiotic pressure, such as methenamine hippurate, vaginal estrogen, and selected non-antibiotic interventions, may support longer-term symptom control [24]. Behavioral strategies should also be integrated into care plans. Although 88.2% reported awareness of hydration advice, only 2.9% identified low fluid intake as a trigger. Counselling should translate this advice into measurable targets, consistent with evidence that increasing daily water intake reduces recurrences in low-fluid premenopausal women [25]. Intercourse-related prevention is especially relevant because 27.8% named sexual intercourse as a precipitant, and sexually active participants were more likely to report this trigger, supporting the use of postcoital prophylaxis and patient-initiated self-start therapy in appropriately selected women [17,18]. Awareness of postcoital voiding was 55.9%, but the evidence for benefit is limited, so expectations should be tempered during counselling [12]. Pelvic floor physiotherapy had been used by 33.3% and is best framed as a supportive adjunct rather than a standalone preventive strategy.

Two additional exploratory findings warrant nuance. Formal education level did not correlate with correct knowledge of the rUTI definition, suggesting that patient education must be specific and clinician-delivered rather than presumed from general education status. In this cohort, routine pre-treatment cultures did not correspond to higher satisfaction, likely reflecting that satisfaction is driven by symptom control and recurrence prevention rather than process measures. Likewise, satisfaction did not differ between women who reported pelvic floor physiotherapy and those who did not (2/12 [16.7%] vs. 3/24 [12.5%]; *p* = 1.00).

Limitations include sample size (N = 36), the single-center, cross-sectional design, reliance on self-report, and use of age ≥50 years as a pragmatic proxy for menopause. The sample was drawn from one Polish urology clinic, which likely introduces selection bias toward specialist-referred, more refractory cases and may restrict generalizability to women managed exclusively in primary care or in other healthcare systems. Demographic breadth was limited, including a relatively small proportion of participants living in large urban areas (22.2%), which may further constrain applicability to more diverse metropolitan populations. Self-reported histories may be subject to recall error and may differentially capture salient past events, which could lead to under- or overestimation of proportions such as ciprofloxacin exposure or urine culture use. Without record linkage, the direction and magnitude of any bias cannot be determined. In addition, physician reasoning, patient-level contraindications, and prior treatment failures were not systematically assessed, limiting our ability to distinguish guideline divergence from appropriate individualized care. These factors limit statistical generalizability; however, the internal consistency of findings across symptoms, behaviors, and care pathways supports their relevance for hypothesis generation and for informing targeted improvements in specialist rUTI management. Finally, regional availability of furazidin may reduce applicability to settings where nitrofurantoin is standard.

### Implications

We propose four practical priorities for clinical improvement in specialist rUTI care. First, standardize patient education around diagnostic criteria, when cultures are recommended, and which prevention strategies have the strongest evidence. Second, implement structured prompts to identify women who may benefit from vaginal estrogen, recognizing that age alone is an imperfect surrogate for menopausal or hypoestrogenic status. Third, strengthen stewardship by prioritizing recommended first-line antibiotics and reducing fluoroquinolone use except when clearly indicated. Fourth, improve the translation of awareness into effective use of non-antibiotic options by offering clear, actionable counselling on methenamine hippurate and cranberry products, including how to choose formulations and set expectations, alongside behavioral targets such as measurable hydration goals and tailored strategies for intercourse-associated recurrences.

## 5. Conclusions

In this specialty-referred cohort of women with recurrent UTI, we observed a high symptomatic burden, frequent disruption of daily functioning, and clear gaps between evidence and practice. Knowledge of core definitions was limited, urine culture use was inconsistent, and exposure to fluoroquinolones remained common despite stewardship guidance. Preventive care was also misaligned: vaginal estrogen, an effective and low-risk option for hypoestrogenic women, was underused, while non-antibiotic strategies showed uneven adoption. Methenamine hippurate use reflected emerging evidence, whereas cranberry products were widely recognized but infrequently implemented; contemporary data support clearer counseling on effective formulations. Behavioral measures, especially specific hydration targets and tailored approaches for intercourse-related recurrences such as postcoital prophylaxis and patient-initiated self-start therapy, merit more systematic integration. Given our small, single-center sample and reliance on self-report, larger prospective studies that link patient-reported outcomes with microbiology and treatment pathways are needed to refine person-centered, evidence-aligned care.

## Figures and Tables

**Table 1:** Demographic and clinical characteristics of the study population (N = 36).

Characteristic	Value
Age, years, mean (SD)	53.0 (14.8)
Age ≥ 50 years, n/N (%)	21/36 (58.3%)
Tertiary education, n/N (%)	17/36 (47.2%)
Urban residence ≥ 100,000, n/N (%)	8/36 (22.2%)
Sexually active, n/N (%)	23/36 (63.9%)
Any contraception, n/N (%)	9/36 (25.0%)
Intercourse reported as trigger, n/N (%)	10/36 (27.8%)
Regular sport, n/N (%)	9/36 (25.0%)
Episodes in last 12 months, median (IQR)	5.0 (4–6)
Most recent episode duration, days, median (IQR)	6 (6.8)
Pre-treatment urine culture, n/N (%)d	14/36 (38.9%)
Post-treatment urine culture, n/N (%)d	5/36 (13.9%)

**Table 2:** Guideline alignment versus reported practices in women with recurrent UTI.

Domain	Guideline-Concordant Care (EAU/AUA)	Reported Practice in This Cohort	Alignment with Guidelines	Key Gap Highlighted
Diagnostic confirmation in recurrent episodes	Obtain urine culture to guide therapy in rUTI episodes and suspected resistance	Pre-treatment culture: 38.9% (14/36); post-treatment culture: 13.9% (5/36)	Low concordance	Limited culture use may reduce diagnostic precision and weaken antimicrobial stewardship
Antibiotic stewardship: agent selection	Prefer first-line agents where appropriate (e.g., nitrofurantoin or nitrofuran alternatives, fosfomycin, and pivmecillinam); restrict fluoroquinolones to selected cases	Prior exposure: ciprofloxacin (55.6% (20/36)); furazidin (75.0% (27/36)); fosfomycin (47.2% (17/36)); pivmecillinam (2.8% (1/36))	Poor concordance	High fluoroquinolone exposure despite guideline recommendations to limit use
Antibiotic duration	Use the shortest effective antibiotic course	Mean reported treatment duration: 9.4 days; 69.4% completed full prescribed course	Partial concordance	Reported durations suggest potential overtreatment in a proportion of cases
Postmenopausal prevention: vaginal estrogen	Recommend vaginal estrogen for rUTI prevention in hypoestrogenic or postmenopausal women	Vaginal estrogen: 5.6% overall (2/36); 9.5% among women ≥50 years (2/21)	Poor concordance	Marked underuse of an effective, low-risk preventive intervention in eligible women
Non-antibiotic prophylaxis: methenamine hippurate	Consider as an evidence-based non-antibiotic preventive option	Methenamine hippurate: 16.7% (6/36)	Partial concordance	Uptake remains limited despite strong evidence supporting its role in reducing antibiotic exposure
Non-antibiotic prophylaxis: cranberry products	May be offered for prevention; benefit depends on formulation and dosing	High awareness (79.4%); use not consistently reported	Partial concordance	Awareness does not consistently translate into evidence-based use
Behavioral strategies: hydration	Encourage adequate fluid intake; increased intake reduces recurrence in selected women	Awareness: 88.2%; low fluid intake identified as trigger: 2.9%	Partial concordance	Advice is widely known but not perceived as a modifiable risk factor
Supportive adjunctive therapies	Pelvic floor physiotherapy may be used as a supportive adjunct	Pelvic floor physiotherapy: 33.3% (12/36)	Good concordance	Appropriate use when framed as adjunctive rather than standalone prevention

## Data Availability

All supporting data generated or analyzed during this study are available from the corresponding author upon reasonable request.

## Supplementary Materials

The following supporting information can be downloaded at https://www.mdpi.com/article/10.3390/medicina62020295/s1: Table S1: English-language version of the questionnaire used to assess knowledge, symptoms, self-care practices, and clinical behaviors in women with recurrent urinary tract infections.

## Abbreviations

The following abbreviations were used in this manuscript:

AUA	American Urological Association
EAU	European Association of Urology
FDA	Food and Drug Administration
IQR	Interquartile range
LUTS	Lower urinary tract symptoms
QoL	Quality of life
UTI	Urinary tract infection
rUTI	Recurrent urinary tract infection
SD	Standard deviation
