# Supplementary material for: From Awareness to Action: Women’s Self-Care Strategies and Clinical Behaviors in Recurrent Urinary Tract Infections
Source: Medicina (Kaunas). 2026 Feb 2;62(2):295. doi: 10.3390/medicina62020295 (PMC12942262; doi:10.3390/medicina62020295)
Supplement: Supplementary file 1 [file medicina-62-00295-s001.zip › medicina-4112081-supplementary.pdf]

Dear Madam,

We kindly invite you to take part in a scientific study that involves completing a short questionnaire about bladder inflammation (cystitis). The questionnaire aims to assess your knowledge of this condition, previous treatments, and experienced symptoms.

The results of the study will help improve future treatment and alleviate symptoms of bladder inflammation. The questionnaire is anonymous, and all data will be used solely for scientific purposes.

GENERAL QUESTIONS

1. How old are you?

____

2. What is your level of education?

▢ Primary education

▢ Secondary education

▢ Higher education

3. What is your current occupation

▢ White-collar work

▢ Manual labor

▢ Unemployed / retired

4. What is your place of residence?

▢ Rural area

▢ Town/city with up to 50,000 inhabitants

▢ Town/city with 50,000–100,000 inhabitants

▢ Town/city with 100,000–500,000 inhabitants

▢ City with more than 500,000 inhabitants

5. Do you suffer from any of the following conditions?

▢ Bruxism

▢ Tension-type headaches

▢ Depression

▢ Autoimmune disease (e.g., Hashimoto's disease, psoriasis)

▢ Stress urinary incontinence

▢ I have allergies (if yes, please specify: ____________________)

6. Are you sexually active?

▢ Yes, I have a regular partner

▢ Yes, I do not have a regular partner

▢ No

7. Have you ever had a bladder infection (cystitis)?

▢ Yes, repeatedly

▢ Yes, occasionally

▢ Yes, once

▢ No

8. Have you given birth? If yes, please indicate the number and way of delivery.

▢ No

▢ Yes, vaginal delivery ___________ times

▢ Yes, cesarean section ___________ times

9. Do you engage in regular physical activity?

▢ Yes

▢ No

10. Do you use any form of contraception?

▢ Yes, hormonal methods (oral contraceptives, intrauterine device, coil)

▢ Yes, mechanical methods (condom)

▢ Yes, spermicidal agents

▢ No

QUESTIONS ABOUT KNOWLEDGE

1. How often must a bladder infection occur to meet the definition of "recurrent"?

▢ Once a year

▢ Three times a year

▢ Six times a year

▢ Once every month

2. The most common cause of bladder infections is:

▢ Bacteria

▢ Viruses

▢ Fungi

3. Which factors increase the risk of bladder infections? Please select all that apply.

▢ Frequent sexual intercourse

▢ Poor hygiene

▢ Wearing tight clothing

▢ Diabetes

▢ Urinary incontinence

▢ Frequent urination

▢ Smoking

▢ Obesity

▢ Use of sanitary pads

4. What are non-pharmacological methods of preventing recurrent infections? Please select all methods you have heard of.

▢ Drinking large amounts of water

▢ Regular urination

▢ Wearing cotton underwear

▢ Bathing in hot water

▢ Wiping from front to back

▢ Urinating immediately after sexual intercourse

▢ Using products/supplements containing cranberry

▢ Urogynecological physiotherapy – pelvic floor muscle exercises

5. How would you rate your knowledge about bladder infections?

▢ Very good

▢ Good

▢ Average

▢ Poor

QUESTIONS ABOUT SYMPTOMS

1. How many times in the past year have you experienced a bladder infection?

____

2. What are the most characteristic symptoms of bladder infection that you experience? Please select all that apply.

▢ Pain or burning during urination

▢ Frequent urination in small amounts

▢ Lower abdominal pain

▢ Cloudy or bloody urine

▢ Fever

▢ Unpleasant urine odor

▢ Pain in the lower back (lumbar region)

▢ I have not had a bladder infection

▢ Other: ____

3. Which circumstances trigger or worsen bladder infections in your case? Please select all that apply.

▢ Menstruation

▢ Sexual intercourse

▢ Exposure to cold (e.g., cold feet)

▢ After using a swimming pool or bathing

▢ Urinating in a body of water

▢ Other: ____

4. Which circumstances trigger or worsen bladder infections in your case? Please select all that apply.

▢ Menstruation

▢ Sexual intercourse

▢ Exposure to cold (e.g., cold feet)

▢ After using a swimming pool or bathing

▢ Urinating in a body of water

▢ Other: ____

5. How many days did your most recent bladder infection last?

_______ days

▢ I have not had a bladder infection

▢ Symptoms persist despite treatment

6. What were the results of your urine tests during your most recent bladder infection? Please select all that apply.

▢ Positive urine culture

▢ Hematuria / erythrocyturia (red blood cells in urine)

▢ Leukocytes in urine

▢ Proteinuria

▢ I have not had a bladder infection

7. During which season are your symptoms most severe or most likely to recur? Please select all that apply.

▢ Spring

▢ Summer

▢ Autumn

▢ Winter

▢ I have not noticed any association with the season

QUESTIONS ABOUT TREATMENT

1. Which antibacterial medications do you use / have you used? Please select all that apply.

▢ Furazidin (e.g., Furagin™)

▢ Fosfomycin (e.g., Monural™, Symural™)

▢ Ciprofloxacin (e.g., Cipronex™, Cipropol™)

▢ Trimethoprim + sulfamethoxazole (e.g., Biseptol™, Bactrim™)

▢ Cefuroxime (e.g., Zinnat™, Zinnox™, Bioracef™)

▢ Pivmecillinam (e.g., X-systo™)

▢ Amoxicillin + clavulanic acid (e.g., Augmentin™, Amoksiklav™)

▢ Cefixime (e.g., Cetix™)

▢ Methenamine (e.g., Urosal™)

▢ I have not used antibiotics

2. Did you experience any side effects after antibiotic therapy?

▢ Yes, gastrointestinal problems

▢ Other: __________

▢ I did not experience any side effects from the medications

3. Which antibiotic did you tolerate the worst?

____

4. Which of the above medications do you consider the most effective?

____

5. How many days did treatment for bladder infection usually last?

____

6. Did you routinely take antibiotics as prescribed, i.e., for the recommended number of days?

▢ Yes

▢ No

7. Do you routinely have a urine culture performed before starting treatment?

▢ Yes

▢ No

8. Do you have a urine culture performed after treatment?
▢ Yes — if yes, how many days after completing treatment? ________________
▢ No

9. Have you ever undergone cystoscopy (bladder endoscopy)?

▢ Yes

▢ No

10. Which antibacterial prophylaxis methods have you used? Please select all that apply.
Please underline the methods you have heard of.

▢ Trimethoprim + sulfamethoxazole (e.g., Biseptol™, Bactrim™) 480 mg after sexual intercourse
▢ Fosfomycin — 1 sachet (e.g., Monural™) every 10–15 days
▢ Furazidin (e.g., Furagin™) 50 mg, 3×2 tablets for 5 days each month
▢ Bacterial vaccine (e.g., Uro-Vaxom™) 1× daily for 3 months
▢ Furazidin (e.g., Furagin™) 2×2 tablets long-term
▢ Vaginal estrogen suppositories in postmenopausal women (e.g., Oekolp forte™)
▢ Prebiotics/probiotics administered orally or vaginally (e.g., Trivagin™, UroLact™, Invag™)
▢ I have not used any
▢ I am not familiar with the above prophylactic methods

11. Have you used any of the following medications that inhibit bladder activity?

Please select all that apply and underline the medication you found most effective.

▢ Solifenacin (e.g., Vesicare™, Solinco™, Vesisol™)

▢ Mirabegron (e.g., Betmiga™)

▢ Oxybutynin (e.g., Driptane™, Ditropan™)

▢ Tolterodine (e.g., Urimper™, Uroflow™, Defur™)

▢ I have not used any of the above medications

12. Have you used hyaluronic acid bladder instillations?

▢ Yes

▢ No

If yes, how would you rate their effectiveness?

▢ Very good

▢ Good

▢ Average

▢ Poor

13. Have you used pelvic floor muscle exercises (urogynecological physiotherapy)?

▢ Yes

▢ No

If yes, how would you rate their effectiveness?

▢ Very good

▢ Good

▢ Average

▢ Poor

14. Are you satisfied with the methods used to treat bladder infections?

▢ Very satisfied

▢ Moderately satisfied

▢ Dissatisfied

Thank you very much for completing the questionnaire and for your time.
